# Emphysematous cholecystitis presenting as gas-forming liver abscess and pneumoperitoneum in a dialysis patient: a case report and review of the literature

**DOI:** 10.1186/s12882-016-0237-3

**Published:** 2016-03-01

**Authors:** Chen-Yi Liao, Chi-chang Tsai, Wu-Hsien Kuo, Ren-Jy Ben, Ho-Cheng Lin, Ching-Chang Lee, Kuan-Jen Su, Han-En Wang, Chih-Chiang Wang, I-Hung Chen, Shang-Tao Chien, Ming-Kai Tsai

**Affiliations:** Department of Medicine, Kaohsiung Armed Forces General Hospital, No.2, Zhongzheng 1st Rd, Lingya Dist, Kaohsiung City, 802 Taiwan R.O.C; Department of Pathology, Kaohsiung Armed Forces General Hospital, Kaohsiung, Taiwan R.O.C; Department of Internal medicine, Division of Nephrology, Tri-service general hospital, National defense Medical center, No.325, Section 2, Cheng-Kung Road, Neihu 114, Taipei, Taiwan R.O.C

**Keywords:** Emphysematous cholecystitis, Gas-forming liver abscess, Pneumoperitoneum, Dialysis

## Abstract

**Background:**

Emphysematous cholecystitis is a rare variant of acute cholecystitis with a high mortality rate. The combination of emphysematous cholecystitis, liver abscess and pneumoperitoneum are even rarer. Herein we present a case of emphysematous cholecystitis in a senile diabetic lady who had worsening hemodynamics while undergoing hemodialysis.

**Case presentation:**

A 64-year-old woman with history of type 2 diabetes mellitus and end stage renal disease with regular hemodialysis presented to the emergency department with a 1-day history of sudden onset of lassitude and hypotension during hemodialysis. The result of a computed tomography (CT)-scan revealed air encircling the gallbladder, liver parenchymal and minimal pneumoperitoneal and liver abscess with no cholelithiasis. The patient had received empirical antibiotics with piperacillin-tazobactam 2.25 g intravenous route every 6 h for 14 days and cholecystectomy with surgical debridement and lead an uneventful postoperative hospital course. Escherichia coli was demonstrated as well as blood culture and peritoneal fluid culture.

**Conclusion:**

In a senile diabetic and dialysis patient, we should take emphysematous cholecystitis into consideration once vague abdominal pain occurrs. Empirical antibiotic therapy and adequate surgical intervention should take place as soon as possible.

## Background

Emphysematous cholecystitis (EC) is a rare life-threatening form of acute cholecystitis representing between 1 and 3 % of acute cholecystitis presenting mainly in male patients aged 50–70 years, and mostly occurring in patients with diabetes mellitus, immunosuppressed and peripheral vascular disease [1].

EC has been characterized clinically by the imaging with gas in the gallbladder lumen, the gallbladder wall and adjacent structure, and elsewhere in the biliary tracts in the absence of an abnormal communication with the gastrointestinal tract.

The gas may disseminate to subcutaneous tissue, as well as to the peritoneal and retroperitoneal cavity. The combination of emphysematous cholecystitis, liver abscess and pneumoperitoneum are rarely seen. Dialysis patients rarely develop such complications according to the review of the literature from Pubmed [2].

Subhepatic abscess involved associated with emphysematous cholecystitis is rare [3–5].

Emphysematous cholecystitis occurring in association with a pneumoperitoneum is relatively rare [6, 7]. A review of the literature from pubmed revealed 18 other cases of this combination and this is the first reported case occurring during dialysis.

Herein we present a dialysis case with clinical image composed of emphysematous cholecystitis, liver abscess and pneumoperitoneum.

## Case presentation

**Fig. 1:**
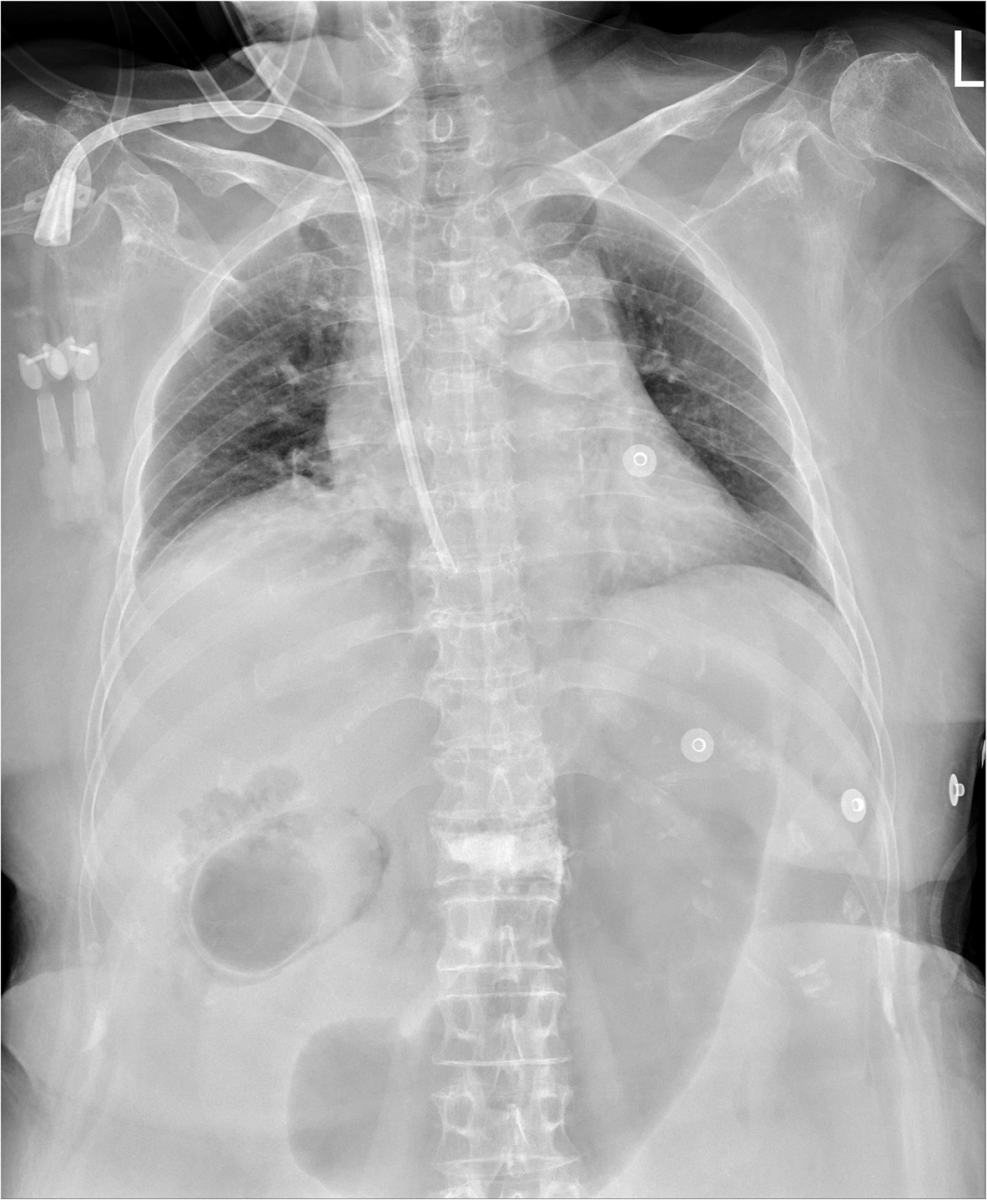
Plain-film radiography showing air in the lumen and wall of the enlarged gallbladder of a 64-year-old woman with abdomen pain and shock while undergoing hemodialysis (arrows)

**Fig. 2:**
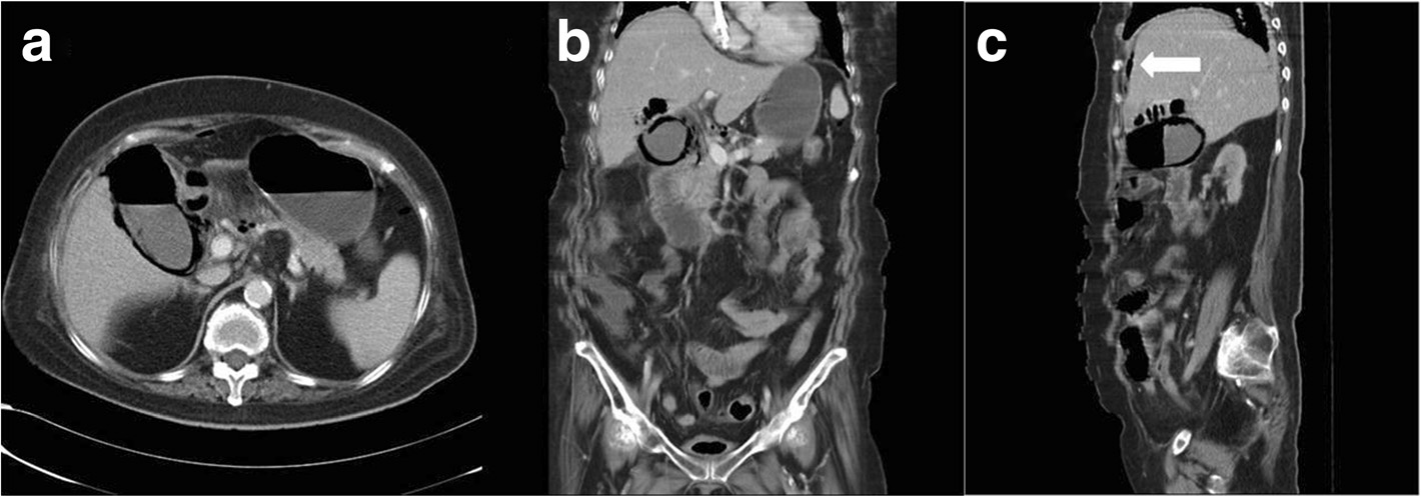
**a** Computed tomography revealed air encircled the gall-bladder lumen as well as intramural and pericholecystic air pockets, with findings pathognomonic for emphysematous cholecystitis. **b** Liver parenchymal destruction by air and partial liver abscess denoted. **c** Pneumoperitoneum as denoted by white arrow

**Fig. 3:**
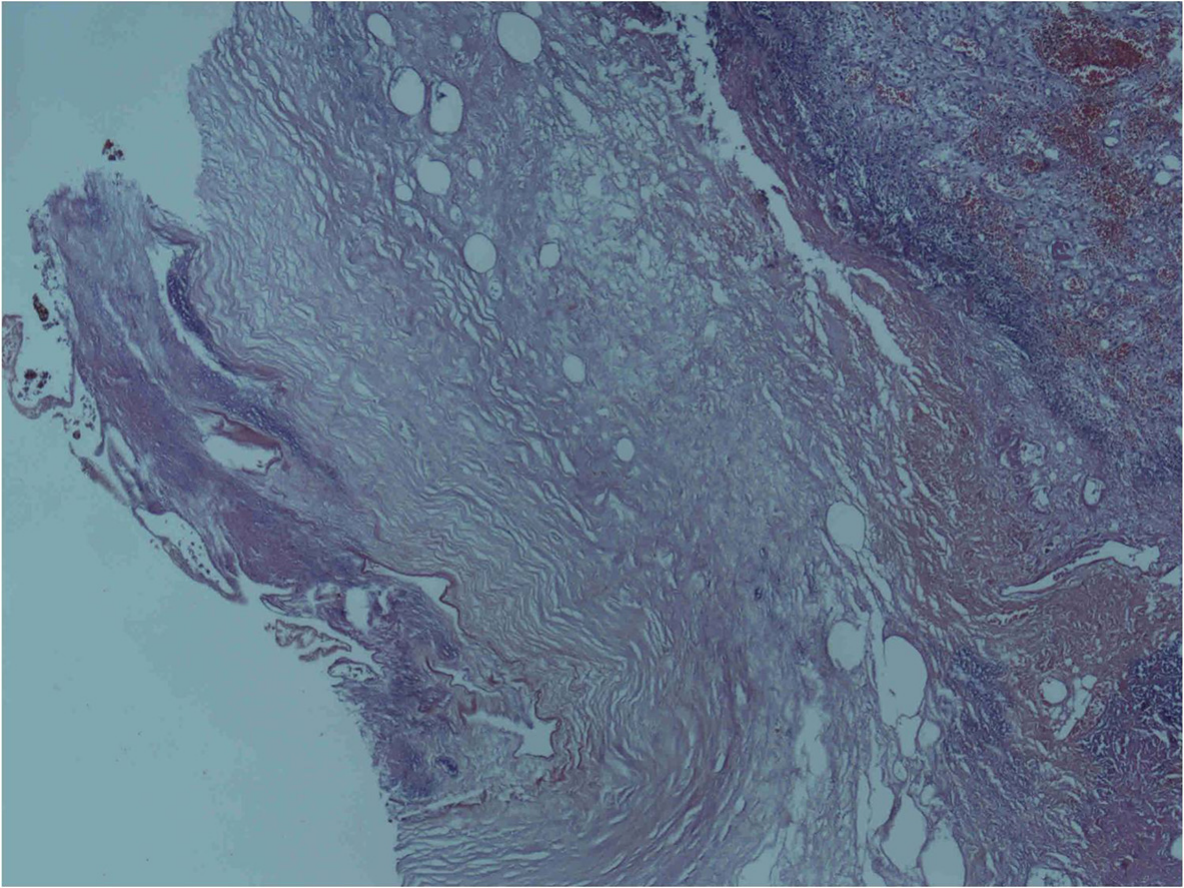
Pathologic analysis of the resected gallbladder disclosed empyema with extensive transmural necrosis and neutrophils infiltration of the whole organ. Mucosa slough with bacteria colonies been observed (arrow)

A 64-year-old woman with history of type 2 diabetes mellitus and end stage renal disease with regular hemodialysis presented to the emergency department with a 1-day history of sudden onset of lassitude and hypotension during hemodialysis. She complained of fluctuating and persistent dull pain over the epigastric area. The painful sensation could not be relieved by lying down or adopting the decubitus position. She did not have nausea, vomiting, tea color urine, clay-like stool, muscle spasm or focal neurologic signs. She denied contact with animals or travel to foreign countries in recent days. On physical examination, the patient was actually ill and had a body temperature of 37.3 °C, pulse rate of 110 beats per minute, respiratory rate of 40 times per minute and blood pressure of 87/65 mmHg. The abdomen revealed right upper quadrant tenderness with Murphy’s sign and muscular defense of the upper abdomen. In reviewing of the system, no diarrhea, no melena or hematochezia, no dysuria, no hematuria, no flank pain, no periumbilical and flank ecchymosis/petechiae been found. In addition, the laboratory examinations revealed leukocytosis (12800/uL) with a left shift (90.4 % neutrophil), elevated C-reactive protein (44.92 mg/dL), liver function impairment (aspartate aminotransferase (AST) of 237 U/L and alanine aminotransferase (ALT) of 232 U/L) and mild jaundice (total bilirubin:1.48 mg/dL). A plain radiography of the chest with the patient in a supine position suggested the presence of a dilated gallbladder with air in the lumen and wall (Fig. 1). The result of a computed tomography (CT)-scan revealed air encircling the gallbladder, liver parenchymal and minimal pneumoperitoneal and liver abscess (Fig. 2a, b, c) with no cholelithiasis. The patient had received empirical antibiotics with piperacillin-tazobactam 2.25 g intravenous route every 6 h for 14 days. The blood culture yield Escherichia coli on the 4th admission day. A general surgeon was consulted and cholecystectomy and surgical debridement performed. The postoperative course went smoothly without any complications . The gallbladder was found to be necrotic. The culture of the bile collected during the operation and the peritoneal fluid collected from the pneumoperitoneum were the same as the blood culture yielded Escherichia coli. Pathologic analysis of the resected gallbladder disclosed empyema with extensive transmural necrosis and neutrophils infiltration of the whole organ (Fig. 3). The patient had developed acute delirium status with response to antipsychotic medication and active upper gastrointestinal tract bleeding with response to proton pump inhibitor therapy during the latter hospital course. She successfully recovered without any sequelae after adequate antibiotic treatement.

## Discussion

EC, also known as acute gaseous cholecystitis is pathophysiologically different from acute or chronic cholecystitis. Obstruction of the gallbladder neck secondary to cholelithiasis induces acute and chronic cholecystitis. EC frequently results from thrombosis or occlusion of the cystic artery with ischemia necrosis of the gallbladder wall with subsequent gallbladder necrosis and secondary infection by gas-forming organisms. EC can be subclassified into 3 differente variants including gas in the gallbladder lumen, gallbladder wall and pericholecystic tissues.

The mortality rate in EC is as high as 15 % compared with 4 % in acute choleycystitis [8–11]. The most common symptoms in EC are right upper quadrant abdominal pain. Fever, nausea and vomiting are also the main clinical symptoms of emphysematous cholecystitis. However, the presenting symptoms of EC are sometimes very vague and initially indistinquishable from those of uncomplicated acute cholecystitis, frequently causing a diagnostic dilemma, as in our patient initially masquerading as hypotension while performing hemodialysis. The symptoms may be even trivial in patients with diabetes mellitus and end-stage renal disease [2, 12].

We had conduct a systemic review from the series literatures of Pubmed with the linkage between “emphysematous cholecystitis”, “diabetes mellitus” and “hemodialysis” which enclosed 23 papers, which include 29 cases of EC, of which 21 cases have the cormobilities of diabetes, one with regular CAPD and the other one received temporary hemodialysis due to acute kidney injury with anuria resulted from hemolytic uremia syndrome. (Table 1) To the best of the author’s knowledge, concurrent EC, liver abscess and pneumoperitoneum in a dialysis diabetic patient has not been previously reported (Table 2). Diabetes usually provide an environment for submucosa thrombosis of biliary tract and predispose patients to fulminant infections. The reason for delay in diagnosis probably involves undetermined diabetes neuropathy, which sometimes masks the symptoms of acute abdomen. Hyperglycemia and ischemia environments in diabetic patients can lead to reduced mobility of phagocytes in the areas of infection and further reduce antimicrobial activity, making EC possible. Appropriate control of blood sugar levels can lower the chance of bacterial overgrowth and associated severity of the disease [11].

**Table 1:** Review of the literature (1955–2015) of emphysematous cholecystitis with association without diabetes mellitus

Number	Age	Gender	Cormobilities	Diabetes	GB stone	Operation /survival	Bacteria source /Concurrent disease /Causative agents other than bacteria	Author (year)
1	54	M	N/A	−	Yes	Yes (Ce)/Yes	B/C: C baratii/Liver abscess/-	Huang et al. (2012) [5]
2	80	F		−	−	PTGBD/-	Bi/C & B/C & abd soft tissue : *Clostridium difficile*/myonecrosis/-	Safioleas et al. (2007) [26]
3	47	M	Alcoholism	−	−	Yes (Ce)/Yes	Bi/C: *Escherichia Coli* & *Enterobacter* Femeral tissue culture: Escherichia Coli,* Bacteroids *&* Enterobacter*/myonecrosis -/ -	Safioleas et al. (2007) [26]
4	72	M	N/A	−	−	Yes (L)/Yes	N/A	Ise et al. (2002) [36]
5	67	F	−	−	−	Yes (Ce)/Yes	B/C, Bi/C negative/Serum: antibodies against Escherichia coli O157,adult-onset HUS; liver abscess/-	Yoshida et al. (1998) [37]
6	41	M	ESRD secondary to Fabry’s with regular CAPD; status post two living related donor transplants; Abdomen vessel calcifications	−	−	Yes (Ce)/Yes	S/C: *Clastridium difficile;* P/C: *Clostridium perfringens*/recent massive UGIB/-	Mirza et al. (1997) [2]
7	64	M	−	−	−	Yes (Ce)/Yes	Bi/C: *Streptococcus* group D-/ -	Carvalho et al. (1965) [38]
8	63	M	−	−	N/A	-/Yes	N/A	Tooms et al. (1955) [39]

*M* male, *F* female, *CA* cancer, *PAD* peripheral arterial disease, *CAD* coronary artery disease, *N/A* unkown, −: none, *SAH* subarachnoidal hemorrhage, *ESRD* end-stage renal disease, *HUS* hemolytic-uremic syndrome, *OP* operation, *CAPD* continous ambulatory peritoneal dialysis, *HG* hyperglycemia, *FBG* fasting blood glucose, *GB* gallbladder, *Ce* cholecystectomy, *Co* cholecystostomy, *Cd* choledochotomy, *PTGBD* percutaneous transhepatic gallbladder drainage, *Lo* laparotomy, *Lc* laparoscopy, *Bi/C* bile culture, *B/C* blood culture, *S/C* stool culture, *P/C* peritoneal fluid culture, *APN* acute pylonephritis, *UGIB* upper gastrointestinal tract bleeding

**Table 2:** Review of the literature (1955–2015) of emphysematous cholecystitis with association with diabetes mellitus or dialysis

Number	Age	Gender	Cormobilities	Diabetes	GB stone	Operation/survival	Bacteria source/Concurrent disease/Causative agents other than bacteria	Author (year)
1	65	F	CAD	Yes	−	PTGBD & liver abscess drainge/Yes	B/C: *Clostridium perfringen*/Liver abscess,hemolysis/ -	Cochrane. et al. (2015) [4]
			PAD					
			Previous acute pancreatitis					
2	85	M	CAD	Yes	−	Yes/Yes	Bi/C: *Clostridium perfringens*/-/ -	Mirrakhimov et al. (2014) [20]
3	77	F	Gastric CA	Yes	−	Yes/Yes	-/-/Chemotherapeutic agents	Kuroda et al. (2013) [21]
			Arterial sclerosis					
4	73	M	Nephropathy Schizophrenia	Yes	−	Yes/Yes	Bi/C: negative/*Escherichia Coli* related APN/-	Ogawa et al. (2012) [22]
5	11	M	Obesity	Yes (type1) 1D	−	Yes (Lc)/Yes	Bi/C: *Enterococcus* P/C: *Escherichia Coli*/Secondary appendicitis/ -	Pal et al. (2011) [23]
6	82	F	SAH	HG while admission (FBG:279mg/dL)	−	Yes/Yes	Bi/C: *Clostridium species*/Subarachnoidal hemorrhage/ -	Uesaka et al. (2009) [24]
7	65	M	Hypertension	Yes	multiple GB stones	Yes (subtotal Ce & Co) /Yes	Bi/C:toxin A of Clostridium difficile and *Escherichia Coli* -/ -	Theodossis et al. (2008) [25]
8	87	F	Bedridden state	Yes	−	PTGBD/-	Bi/C & B/C & abdomen soft tissue culture: *Clostridium difficile*/Renal failure; myonecrosis/-	Safioleas et al. (2007) [26]
9	32	M		Yes	−	Yes (Ce)/No	Bi/C: *Escherichia Coli* & *Clostridium Welchii*; Femeral tissue culture: *Escherichia Coli*/Myonecrosis-	Safioleas et al. (2007) [26]
10	70	M	Heart disease Hyperlipidemia	Yes	Multiple small GB stones	Yes (Ce)/Yes	Bi/C: *Clostridium* perfringens-/ -	Shresth et al. (2007) [27]
11	68	M	Hypertension CAD	Type 1	−	Yes (Ce)/Yes	B/C: *Clostridium perfringens* and *Corynebacteria*-/ -	Bernstein et al. (2007) [28]
12	64	F	Hypertension	Yes	Yes	Yes (Ce)/Yes	B/C: Salmonella derby Bi/C: Negative-/ -	Moanna et al. (2006) [11]
			Hypothyroidism					
			Anemia					
13	62	M	Alcoholism	Yes	−	Yes (Ce)/Yes	Bi/C: *Klebsiella pneumonia*-/ -	Prieto Fernández et al. (2004) [29]
			Atrial fribrillation					
14	62	M	Alcoholism	Yes	−	Yes (Ce)/Yes	Bi/C: *Klebsiella pneumonia*-/	Prieto Fernández et al. (2004) [29]
			Overweight				−	
			Atrial fribrillation					
15	42	M	Recurrent UTI	Yes	Yes	Yes (Ce)/Yes	U/C & Bi/C : Negative /Emphysematous pyelonephritis/-	Bhansali et al. (2004) [30]
16	55	M	Hypertension	Yes	Yes	-/Yes	N/A	Chiu et al. (2004) [31]
17	70	M	N/A	Yes	Yes (Mirizzi syndrome)	Yes (Ce)/Yes	N/A	Ozkan et al. (2003) [32]
18	66	F	Gastric CA post OP	Yes	−	Yes (PTGBD; Ce and Cd) /Yes	Bi/C: Clostridium perfingens & E. coli/pneumobilia/-	Ohtani et al. (1996) [33]
			Breast CA post OP					
19	77	M	−	Yes	−	N/A	N/A /Liver abscess-	Matsura et al. (1995) [34]
20	66	M	−	Yes	Yes	Yes (Ce)/Yes	N/A-/ -	Carvalho et al. (1965) [35]
21	68	M	−	Yes	N/A	-/N/A	- /-/ -	Carvalho et al. (1965) [35]

*M* male, *F* female, *CA* cancer, *PAD* peripheral arterial disease, *CAD* coronary artery disease, *N/A* unkown, −: none, *SAH* subarachnoidal hemorrhage, *ESRD* end-stage renal disease, *HUS* hemolytic-uremic syndrome, *OP* operation, *CAPD* continous ambulatory peritoneal dialysis, *HG* hyperglycemia, *FBG* fasting blood glucose, *GB* gallbladder, *Ce* cholecystectomy, *Co* cholecystostomy, *Cd* choledochotomy, *PTGBD* percutaneous transhepatic gallbladder drainage, *Lo* laparotomy, *Lc* laparoscopy, *Bi/C* bile culture, *B/C* blood culture, *S/C* stool culture, *P/C* peritoneal fluid culture, *APN* acute pylonephritis, *UGIB* upper gastrointestinal tract bleeding

Chen et al. reported that end-stage renal disease was an independent risk factor for acute cholecystitis. The independent risk factors were older age, higher Charlson's score, atrial fibrillation, severe liver disease, diabetes, and dialysis modality. Haemodialysis patients had a higher risk of acute cholecystitis than PD patients [12].

Another possible postulated mechanism in EC is the fluctuating hemodynamic change during hemodialysis compared with peritoneal dialysis [13]. Hypotension in dialysis patient results from rapid reduction of blood volume owing to ultrafiltration and decrease in extracellular osmolarity during the dialysis session especially in older and diabetic patient with coexisting illnesses, such as cardiovascular diseases, which might contribute to systemic hypoperfusion and further compromise visceral circulation such as the cystic artery which lead to gallbladder ischemia and facilitates the proliferation of gas-forming organisms and bacterial translocation in the devitalized tissue with low oxygen saturation. The hypotension episode in our patient can be overlooked due to underlying bacteremia related septic shock may mimic the presentation of dialysis process. Besides, the inflammation and oxidants produced after ischaemia/reperfusion also impair the emptying of the gallbladder, increase the residual volume, and reduce smooth muscle contractility, which may increase the rate of acute cholecystitis in HD patients [14]. Microinflammation in hemodialysis patients also could lead to gut bacterial translocation which further aggravats EC [15]. Chen at al proposed that uremic toxin and increased oxidative stress are both predisposing factors for causing increased gallbladder mucosa inflammation and irritation which further contributed to acute cholecystitis in ESRD patients [12]. The pathogens responsible for the gas formed in EC are usually anaerobes like Clostridium spp, or other microorganisms like Escherichia coli, and Klebsiella spp,Proteus culgaris, Aerobacter aerogenes, Staphylococcus, Streptococcus, and Salmonella derby [11].

Our case demonstrates Escherichia coli in bile culture, blood culture and peritoneal fluid, which suggested that the hemodynamic instability during dialysis favored results from disseminated infection with septic shock. Escherichia coli is a common bacteria present in the gastrointestinal tract, especially in the colon and small bowel. Bactereia tranlocate from duodenal to the biliary tract when local aggressive factors take place, such as peptic ulcer or hemodialysis in our patient [16]. CT scanning of the abdomen is the most sensitive technique for diagnosing emphysematous cholecystitis by presence of gas within the gallbladder wall and lumen. The clinical picture in our case demonstrated liver abscess accompanied with gas retention in the liver parenchyma, gallbladder and biliary tract. Delay in diagnosis of EC could lead to liver abscess formation [5, 15]. The presence of a concomitant pneumoperitoneum, which may occur following gallbladder perforation, is rarely found. Most patients with a concomitant pneumoperitoneum are an emergent condition that requires emergency exploratory laparotomy, followed by cholecystectomy as in our case. Another method of treatement, involves initial percutaneous cholecystostomy with a strict intravenous antibiotics regimen, followed by subsequent cholecystectomy during second stage. In severely ill patients in particular, percutaneous cholecystostomy with broad –spectrum antibiotics may be an alternative choice of treatment.

There are scarce information about the treatment in patients with EC in patients underwent hemodialysis due to end stage renal diseas. Yeh had carried out that laparoscopic cholecystectomy is safe for ESRD patients with gallbladder lesions. Similar blood loss, conversion rate, morbidity, mortality, and hospital stay were achieved by applying laparoscopic cholecystectomy to treat ESRD patients compared with the normal populations [17]. Gunay et al. had proposed that in a patient underwent hemodiaysis with concurrent acute choleycystitis, cholecystectomy may be a better initial choice compared with percutaneous cholecystostomy due to higher success rate and lower morbidity and mortality rate [18]. Gumus et al. suggested that in the management of acute cholecystitis patients with chronic hemodialysis states especially in poor surgical candidate, percutaneous cholecystostomy may be the alternative choice [19].

## Conclusions

In conclusion,emphysematous cholecystitis is a rare form of cholecystitis especially in dialysis patients which could be fatal if delayed in diagnosis and progress to pneumoperitoneal and liver abscess. In a senile diabetic and dialysis patient, we should take emphysematous cholecystitis into consideration once vague abdominal pain and hypotension occurs in a patient underwent hemodialysis. Empirical antibiotic therapy and adequate surgical intervention should take place as soon as possible.

## Consent

Written informed consent was obtained from the patient for publication of this Case report and any accompanying images. A copy of the written consent is available for review by the Editor of this journal.

## Abbreviations

ALT: alanine aminotransferase
AST: aspartate aminotransferase
CT: computed tomography
EC: emphysematous cholecystitis
